# Repeated surveying over 6 years reveals that fine-scale habitat variables are key to tropical mountain ant assemblage composition and functional diversity

**DOI:** 10.1038/s41598-020-80077-8

**Published:** 2021-01-08

**Authors:** Mulalo M. Muluvhahothe, Grant S. Joseph, Colleen L. Seymour, Thinandavha C. Munyai, Stefan H. Foord

**Affiliations:** 1 SARChI-Chair On Biodiversity Value and Change, Department of Zoology and Centre for Invasion Biology, School of Mathematical and Natural Science, University of Venda, Private Bag X5050, Thohoyandou, 0950 South Africa; 2 DST/NRF Centre of Excellence, Percy FitzPatrick Institute of African Ornithology, Department of Biological Sciences, University of Cape Town, Rondebosch, 7701 South Africa; 3 South African National Biodiversity Institute, Kirstenbosch Research Centre, Private Bag X7, Claremont, 7735 South Africa; 4 School of Life Science, College of Agriculture, Engineering and Science, University of KwaZulu-Natal, Private Bag X01, Scottsville, 3209 South Africa

**Keywords:** Ecology, Biodiversity, Climate-change ecology, Community ecology, Conservation biology, Tropical ecology

## Abstract

High-altitude-adapted ectotherms can escape competition from dominant species by tolerating low temperatures at cooler elevations, but climate change is eroding such advantages. Studies evaluating broad-scale impacts of global change for high-altitude organisms often overlook the mitigating role of biotic factors. Yet, at fine spatial-scales, vegetation-associated microclimates provide refuges from climatic extremes. Using one of the largest standardised data sets collected to date, we tested how ant species composition and functional diversity (i.e., the range and value of species traits found within assemblages) respond to large-scale abiotic factors (altitude, aspect), and fine-scale factors (vegetation, soil structure) along an elevational gradient in tropical Africa. Altitude emerged as the principal factor explaining species composition. Analysis of nestedness and turnover components of beta diversity indicated that ant assemblages are specific to each elevation, so species are not filtered out but replaced with new species as elevation increases. Similarity of assemblages over time (assessed using beta decay) did not change significantly at low and mid elevations but declined at the highest elevations. Assemblages also differed between northern and southern mountain aspects, although at highest elevations, composition was restricted to a set of species found on both aspects. Functional diversity was not explained by large scale variables like elevation, but by factors associated with elevation that operate at fine scales (i.e., temperature and habitat structure). Our findings highlight the significance of fine-scale variables in predicting organisms’ responses to changing temperature, offering management possibilities that might dilute climate change impacts, and caution when predicting assemblage responses using climate models, alone.

## Introduction

The signature of climate change is now apparent in changes in species’ distributions, as species move in response to warming. For example, the median altitudinal range change is 11 m upwards per decade^1^. Mountain-dwelling assemblages often include many endemics adapted to narrow niches, isolated in a landscape matrix that limits dispersal^2–4^. These assemblages are particularly susceptible to climate change, as high-altitude zones should experience faster rates of warming^5^. Upslope migration of organisms into smaller areas^6^, when habitat allows, increases interspecific competition and reduces population sizes^7^. Aspect is also influential, as equator-facing slopes experience greater radiation and higher temperatures than slopes facing away from the equator^8^. These differences in abiotic conditions are reflected in the structure and composition of vegetation on the different mountain aspects, and in species’ distributions and behaviour on mountain slopes^9^. For example, the red ant *Myrmica sabuleti* is primarily limited to south-facing slopes in the United Kingdom, where temperatures are sufficiently warm^10^. Shifts in temperature regimes associated with climate change should affect ectotherms, in particular, as they have fewer physiological adaptations for coping with temperature changes than endotherms^11^.

Ants (Hymenoptera: Formicidae) are abundant and found on most continents^12^. Their community composition and role in ecosystem functioning can effect considerable changes in ecosystem structure and function^13–16^. They are considered thermophilic (i.e., heat-loving)^17^, and ant species richness can decrease with altitude (along with decreasing temperature), or exhibit mid-elevational peaks^18,20^. Cool temperatures should limit ant species’ distributional ranges^3^, with some species occupying areas only because they can tolerate lower temperatures than more dominant species^19,20^. Displacement with climate change of cold-adapted species by those that can tolerate warmer temperatures has now been found in two *Aphaenogaster* ant species in the Appalachian Mountains^21^. Different temperature tolerances imply that ant species assemblages should change with increasing elevation to either a subset of species that can tolerate low temperatures, or to a distinct assemblage found only at higher elevation sites, and ant assemblages on the pole-facing aspect of mountains could be similar to those found at higher elevations on the equator-facing side. Alternatively, if ants are more influenced by habitat structure, which may differ between the two aspects, ant assemblages should differ significantly between the two mountain aspects.

Microclimates, for example, those created by topography^22^, vegetation^15,23^ or soils^24^, can modify temperature extremes, buffering against climate change. Microclimates may allow species with different thermal tolerances to coexist, increasing diversity^25^. Thus, any consideration of effects of altitude on species assemblages should include not only the larger-scale measures of altitude and aspect, but also finer scale measures of vegetation and soils, which can also influence ant assemblages^26^. Furthermore, factors like vegetation cover are amenable to management interventions, allowing for some adaptation to climate change^27,28^.

In the Soutpansberg, in tropical north-eastern South Africa, the impact of aspect is considerable: climate differs markedly between the north and southern aspects, owing to the mountain’s East–North–East to West–South–West orientation, resulting in arid slopes on the northern aspect characterised by open dry savanna, and thicket or forest on the mesic southern aspect, with herbaceous habitats at the highest elevations^18,29^. The region is vulnerable to global change, currently experiencing reduced summer rainfall, elevated surface temperatures, and widespread, more frequent droughts^23,30^. Recent models reveal contraction of species’ ranges at higher elevation from montane regions in two southern African countries^4^. In our study site, ants show a decrease in species diversity with increasing altitude^18^, and at higher altitude sites, diversity over time seems to be more variable^31^. Here, we assess whether ant species composition across elevational gradients changes owing to species turnover or species loss, by assessing the relative contribution of these two components to beta diversity over 6 years. We expect total beta diversity for mountain slopes to increase with increasing elevation, and perhaps also with time. Ants have been found to display patterns of species turnover with change in elevation^32,33^, so we expect the species turnover component to represent most beta diversity, with little contribution from species nestedness. We also evaluate whether ant assemblages at different elevations and aspects of the Soutpansberg mountain vary with altitude and/or aspect as well as fine-scale vegetation measures.

Given that species assemblage composition influences the traits of species present and thus the functions they perform, we also investigate patterns in functional diversity with different elevations, vegetation, soil structure and aspect. Functional diversity (FD) is the range and value of species traits present within a community, which influences how those species contribute to ecological function^34^. Thus FD reflects ecosystem pattern and process^35^. With global change, there is growing interest in how FD varies across environmental gradients^33,36,37^. Given that heating could be associated with changes to species composition and the number of species, particularly at altitude^31^, patterns of FD with altitude and aspect may give insights into whether and how the role of ants in ecosystem functioning might change, or whether species are merely replaced by functional analogues along abiotic or biotic gradients. At environmental extremes, resources are increasingly limited, which can amplify environmental filtering^38,39^. Such filtering can select for functionally-unique traits which enable species to cope with extreme conditions, with only species possessing the necessary traits persisting. A pattern of increasingly-nested subsets of available functional strategies with rising altitude has recently been found for ants elsewhere^33^, so we might expect lower FD at higher altitudes.

Most studies are carried out over relatively short time periods, delivering only snapshots of pattern and process. Here, we collected data biannually for six consecutive years, producing one of the largest standardised, spatio-temporal invertebrate assemblage datasets assembled to date. We asked (i) whether ant assemblages at high altitude are subsets of those found at lower altitudes, or whether they are a different set of species, and whether there is evidence of change in beta diversity over the relatively short period of 6 years; (ii) whether ant assemblages respond primarily to abiotic factors like temperature and solar radiation, or primarily to habitat structure, or whether both abiotic and biotic factors influence species composition; and (iii) whether FD displays patterns with elevation, aspect and/or habitat structure.

## Materials and methods

### Study site

Ants were sampled in the Soutpansberg Mountains, Vhembe Biosphere Reserve, a recognised southern African centre of endemism^40^. We sampled 11 sites spaced between 160 to 290 m apart (Electronic Supplementary Material Fig. 1) across an elevational transect (beginning at 23° 02′ 16.91ʺ S, 29° 26′ 34.22ʺ E) running north to south, starting at 800 m above sea level (a.s.l.) on the southern aspect, ascending to 1700 m a.s.l., before descending to 800 m a.s.l. on the northern aspect. Erosion-resistant quartzite, conglomerate, sandstone, shale rocks, and basalt characterise the transect^41^, which has wet summers, dry winters and mean annual precipitation of about 450 mm^42^.

### Ant sampling

Epigaeic ants were sampled each January (wet season) and September (dry season) from 2009 to 2015, at the 11 sites. Each site housed four replicates, spaced 300 horizontal metres apart to avoid pseudo-replication^43^. Each replicate contained ten pitfall traps (ø 62 mm), 10 m apart, in a grid of two parallel lines (2 × 5). Traps containing a 50% solution of propylene glycol were left open for 5 days in each survey. Ants from traps were then collected and identified to species level where possible, otherwise to genus and then morphospecies. Specimens are lodged in the University of Venda Natural History Collection housed in the Chair on Biodiversity Value and Change.

### Vegetation structure sampling

Horizontal habitat (vegetation) structure was quantified using a 1 m^2^ grid placed over each pitfall trap, and percentage area of bare ground, vegetation, rock and leaf litter was calculated. To quantify vertical habitat structure, we used four rods (1.5 m high) counting the number of vegetation contacts recorded along 25 cm intervals of the rod (0–25 cm, 25–50 cm, 50–75 cm, 75–100 cm, 100–125 cm, 125–150 cm, 150 + cm). These four rods were placed 1.5 m away from the pitfall trap, in a straight line originating at the pitfall trap and passing through each corner of the 1 m^2^ grid. To summarize vertical and horizontal habitat structure, we performed a Principal Component Analysis (PCA) on the vegetation measures we had gathered for vegetation structure, to produce two main axes for horizontal (i.e., pch1 and pch2) and vertical (pcv1 and pcv2) vegetation structure.

### Soil sampling

In January 2010, ten soil samples were taken from each replicate using a soil auger, 7.5 cm in diameter and 10–15 cm deep. The ten samples were mixed, dried, and analysed by BemLab (Pty) Ltd laboratories, South Africa for composition (clay, sand, rock and silt), pH, conductivity, C, K, Na, Ca, Mg, P, and NO_3_. We then summarised soil characteristics using a PCA, for which the two main axes were pcsoil1 and pcsoil2.

### Temperature measurement

Within two replicates at each site, one Thermocron iButton (Semiconductor Corporation, Dallas/Maxin TX and USA), buried 1 cm below the soil recorded temperature at hourly intervals for the entire period of sampling. These readings were used to generate maximum (T_max_), mean (T_mean_) and minimum (T_min_) temperature readings for each site and sampling period.

### Functional traits

We measured seven morphological traits (Table 1) relating to resource use by ants^44,45^ using a ZEISS Discovery V12 Modular Stereo Microscope with Motorized 12 × Zoom Module and Zeiss Application Suite V3.024, calibrated using a stage micrometer (Jena, Germany). A minimum of five individuals were measured for each species. Only minor workers were measured. There may be microclimatic variation in species’ sizes, so we aimed to measure specimens from each elevation. For most species, this was not possible, however, as they did not occur across the altitudinal range, or there were too few specimens to assess intraspecific size variation.

**Table 1:** Ant morphological traits that were measured and used to assess functional diversity of ant assemblages, and their functional significance.

Morphological trait	Continuous measures of traits (mm)	Hypothesized functional significance
Mandible length	From the mandibular apex to the anterior clypeal margin	Indicative of diet; longer mandibles allow predation of larger prey^76^
Clypeus length	Maximum length of the clypeus	Well-developed clypeus relates to liquid feeding behaviour^77^
Head width	Maximum width of the head across the eyes	Size of spaces through which ant can pass^78^; mandibular musculature^79^
Eye length	Maximum diameter of the compound eye	Indicates feeding behaviour, predatory ants have proportionately larger eyes^80^
Scape length	Maximum straight-line length of the scape	Related to perception; scape size correlates to simplified environments^80^
Weber’s length	Diagonal length of the mesosoma in profile from the point at which the pronotum meets the cervical shield to the posterior basal angle of the metapleuron	Indicative of body size^54^, correlates to habitat complexity and metabolic function^44^. Larger ants tend to be predatory^81^
Hind femur length	Maximum length of the hind femur	Related to increased foraging speed in simple habitats, smaller leg length allows exploitation of complex habitats^82^

### Data analysis

#### Sampling sufficiency

We compiled sample-based rarefaction curves for ants for each altitude at each aspect using the nonparametric Incidence Coverage Estimator (ICE) and Michaelis–Menten richness estimate to determine adequacy of sampling, in the programme EstimateS^46^. Sampling was deemed adequate when values converged at the highest values (Electronic Supplementary Material Table 1).

#### Ant assemblage beta diversity

We assessed whether ant assemblages at high altitudes were subsets of those found at lower altitudes, or whether they comprised completely different species, and whether there was a signal of change in ant species composition over the 6 years of the study, using nestedness and turnover components of beta diversity^47^. β_sor_ gives the total variation in composition between assemblages, and is the sum of variation owing to nestedness (β_sne_) and species turnover (β_sim_). We first assessed the components of beta diversity on data from traps combined from all years, per site, and then also on sites only on the north and south aspects, using the package “betapart”^48^ on presence-absence data. Then, using presence-absence data for the different dates of sampling, we plotted a dendrogram to allow visualisation of the different plots at different times, clustering using group averaging. This dataset allowed us to assess decay in similarity between assemblages, by fitting a negative exponential function to increasing assemblage dissimilarity over spatial (i.e., altitude within aspect) and temporal (i.e., total days from the first day of sampling) distances. To assess temporal changes in beta diversity, we allocated sites to one of three categories (low: < 1000 m a.s.l., medium: 1000 – 1400 m a.s.l, and high: > 1400 m).

#### Ant assemblage composition

We pooled assemblage data for traps within an elevational band and aspect, but kept these separate for sampling season and year. These multivariate abundance data formed the response variable for generalised linear models (GLM), testing for differences in assemblage composition with altitude, aspect, season and interactions between them, as well as measures of habitat (i.e., horizontal and vertical vegetation and soil structure, minimum, maximum and mean temperatures). We assessed these using the R package “mvabund”^49^ with the functions “manyglm” and “anova.manyglm”, using a negative binomial distribution with log-link. This approach accounts for confounding mean–variance relationships, which are common in abundance data containing many zeros^50^. Likelihood ratio statistics are summed for each species, yielding a community-level measure for each altitude and aspect category, and the PIT-residual bootstrap method^51^ derives p-values by resampling 999 rows of the dataset. Models were checked for departure from model assumptions by visually examining plots of Dunn-Smyth residuals against fitted values to identify any non-random patterns. We also performed an unconstrained ordination, using the package “boral” in R^52^, modelling species abundance data using a negative binomial distribution and plotting the relationships between sites in two dimensions. To help visualise these assemblages, we allocated sites to one of five altitudinal bands, each spanning 180 m. These were: A = 810–990 m; B = 991–1170 m; C = 1171–1350 m; D = 1351–1530 m; E = 1531 m–1710 m; and north (N) and south (S) aspect categories. Thus AS indicates traps within the elevational band of 810 – 990 m a.s.l. on the southern slope, and CN indicates traps between 1171 and 1350 m on the northern aspect.

#### Functional diversity across an altitudinal gradient

We used FD as measured by Petchey and Gaston^53^. Trait data were standardized to ensure biological variation within each trait was treated equally (each trait thus had a mean of zero and a standard deviation of one). We gave Weber’s length a double weighting as it reflects not only habitat complexity and metabolic function, but is indicative of body size (key in thermoregulation^44,54^). We used Gower distance to convert the species by trait matrix to a distance matrix, and clustered the matrix using the “average” method^55^, which produced the highest cophenetic correlation (0.87) between estimated and original distances arising from the dendrogram. Thus, each species was given a measure of similarity with every other species, based on the functional traits used. Adding a species to an assemblage increases the likelihood of adding new traits, so FD tends to increase with species richness^56^. To account for this, simulation models were adopted, comparing observed FDs against a null distribution of FD values. This allows calculation of standardised effect size (sesFD), which corrects for increases in FD with added species, providing a reliable measure of degree of functional differentiation within assemblages (sesFD). We calculated sesFD using the analogous ses.pd function in the R package “picante”^57^. For a site with *n* species, the simulation models randomly select *n* species from the total pool without replacement, and formulate an expected FD for that group of species. We used the independent swap method, in which species number was maintained within each sample, but the number of sites in which each species occurred was kept constant, so that species were only included in simulations proportional to their occurrence in the dataset. We ran models 999 times, creating a distribution of 1000 values for each observed value. The mean of this distribution was then subtracted from the observed FD value and divided by the standard deviation of the null distribution, to provide sesFD. Thus sesFD reflects the number of standard deviations in which the observed community is above or below the mean (0) of the simulated communities from the null model^58^.

To assess how sesFD changed with environmental variables, we used a general linear mixed model, with sesFD as the dependent variable, and altitude, aspect, vegetation (represented by pch1, pch2, pcv1 and pcv2), soils (pcsoil1 and pcsoil2), temperature (T_Max_, T_mean_ and T_Min_), and interactions between altitude and aspect, as explanatory variables, with site as a random intercept variable, using the package nlme^59^ in R statistical programme version 3.0. We included an interaction between altitude and aspect because a lower altitude on the south side could have the same sesFD as a higher altitude on the north side, owing to the effects of aspect on temperature. We used the “dredge” function in MuMIn^60^ to find the best models by specifying that only explanatory variables correlated with Pearson correlation < 0.5 could be included in any single model, and identified the best models as those within two units of that with the lowest AICc, and thus have substantial support of being the best models^61^. We then used model averaging on these models. We calculated variance explained by fixed (marginal R^2^), and fixed and random effects (conditional R^2^, ^62^) for the best and worst models included in the model averaging, and report on both of these. We checked for constant error variance by plotting residuals against fixed values, and quantile–quantile plots to assess normality of errors for these models.

## Results

A total of 102,496 ants representing 35 genera and 122 species were caught in pitfall traps over 6 years of sampling. Of these, 102 species had five or more individuals, and provided reliable, standardised trait data for use in FD analysis. Few species were present in sufficient abundance across sites along the elevational gradient to allow specification of a size per altitude. Furthermore, interspecific variation was greater than intraspecific variation, so we used mean measures for ants across all sites as traits.

### Vegetation structure

For horizontal habitat structure, the first axis (pch1) captured 41%, the second (pch2) 30% of the variation, whilst for vertical habitat structure, these two axes captured 37% (pcv1) and 24% (pcv2) of variation. The first principal component axis for horizontal habitat structure (pch1) correlated positively with bare ground and negatively with vegetation cover. The second (pch2) correlated positively with leaf litter cover and negatively with rock cover (Electronic Supplementary Material Fig. 2a). For vertical habitat structure, pcv1 increased with vertical structure, particularly at levels between 50 and 125 cm. The second axis, pcv2, correlated positively with canopy cover above 100 cm in height, but negatively with cover below 75 cm (Electronic Supplementary Material Fig. 2b).

### Soil characteristics

The PCA for soils explained 61% of the variation, the first axis explaining 46%, the second, 15%. The first principal component axis (pcsoil1) was positively correlated with acidic soil and negatively with basic soils. The second principal component axis (pcsoil2) was positively correlated with sites that had sandy soil and negatively with clay (Electronic Supplementary Material Fig. 3).

### Beta diversity of ant assemblage composition: nestedness or turnover

Beta diversity was primarily driven by species turnover. For north and south sites together, total beta diversity was 0.682, the component explained by nestedness was only 6.1% (0.042), with turnover explaining the remaining 93.9% (0.641). When only sites on the northern aspect were compared, total beta diversity was 0.510, the nestedness component was 0.069 (13.5%), the remaining 86.5% (0.441) was species turnover. On the southern aspect, total beta diversity was similar to that of the northern aspect, at 0.500; the nestedness component was lower as a proportion than for the northern aspect, however, at 0.043 (8.7%) and turnover was 91.3% (0.453).

**Figure 1:**
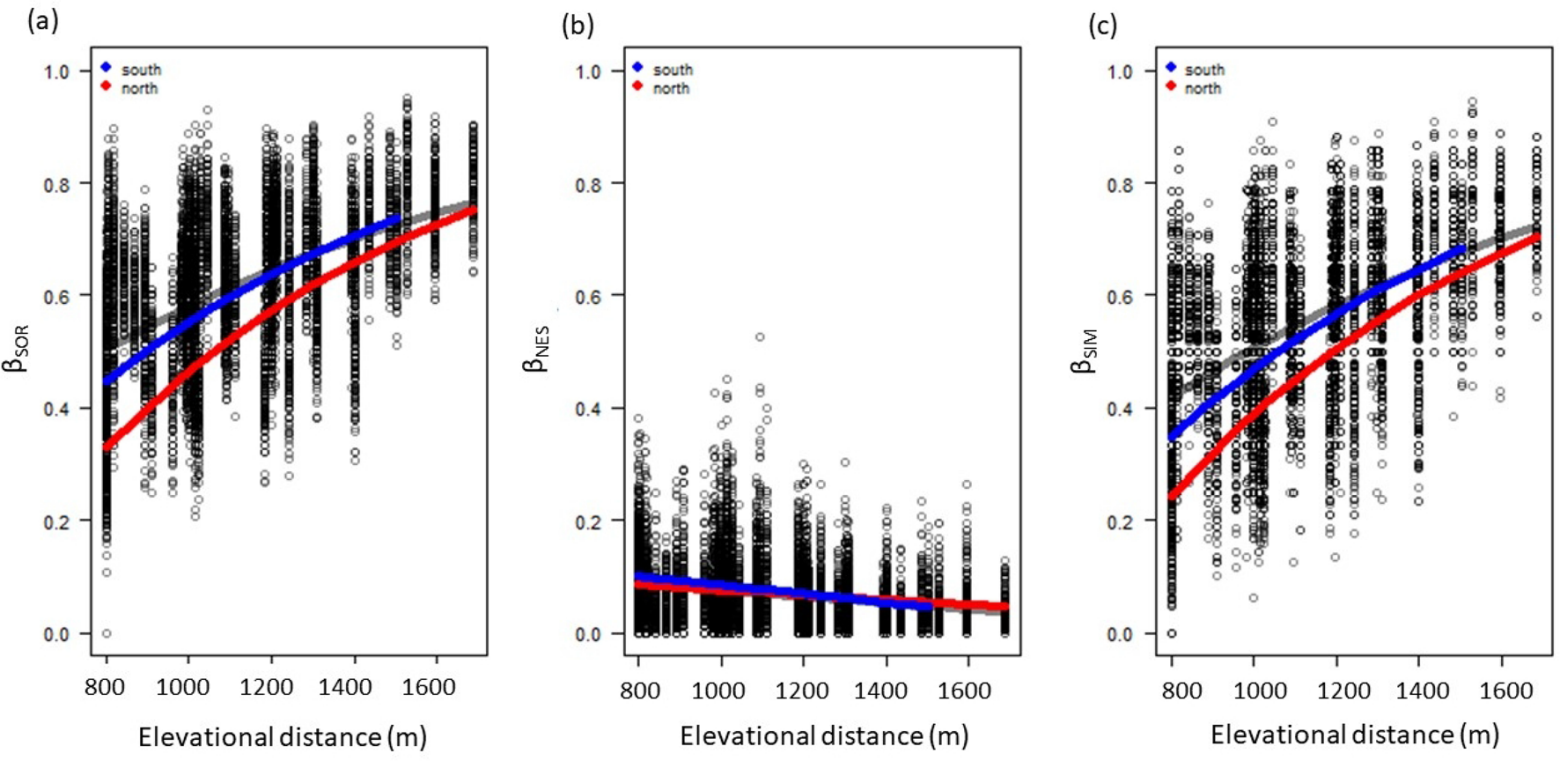
The relationship between ant species (**a**) total beta diversity, β_sor_; comprised of its (**b**) nestedness-resultant component, β_nes_, and (**c**) turnover component, β_sim_ for the Soutpansberg, South Africa. The red and blue lines represent the mixed model predictions for north and south aspects, based on data for all years, with each data point representing a comparison between the lowest elevation sites (810 m) and subsequent higher elevations.

Beta-decay models show increasing total beta diversity, β_sor_ with elevation, although the rate of increase slowed with increasing elevation (Fig. 1a). The trend of increasing β_sor_ over space was statistically significant (β_slope_ = 0.00084, n = 8645, pseudo r^2^ = 0.26, p = 0.01). The component of beta diversity owing to nestedness declined (Fig. 1b), whilst that associated with turnover increased (Fig. 1c), with altitude.

A comparison of beta decay over time found no significant patterns for low (p = 0.78) or medium (p = 0.61) altitude ant communities over the 6 years of our study, but there was a significant signal of change in beta diversity over time for high altitude sites, although the explanatory power was low (β_slope_ = 5.4 × 10^–5^, n = 1127, pseudo r^2^ = 0.01, p = 0.01, Electronic supplementary Fig. 4).

### Ant assemblages relative to aspect and known altitudinal thresholds

**Figure 2:**
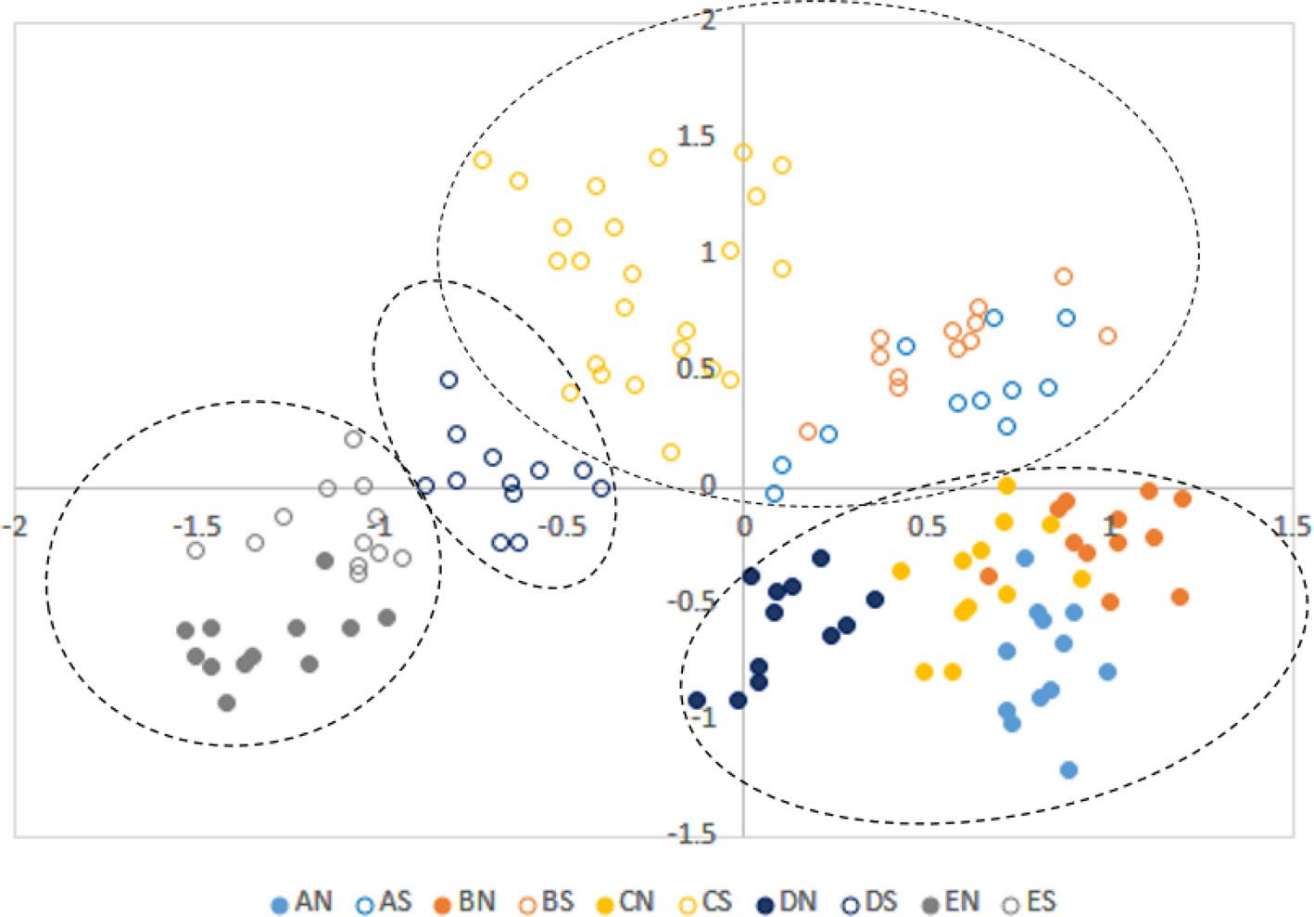
Ordination representing ant species assemblages relative to altitude (Categories: A = 810–990 m; B = 991–1170 m; C = 1171–1350 m; D = 1351–1530 m; E = 1531 m–1710 m) and aspect (*N* northern, *S* southern). Dashed groupings represent assemblages similar at 30% or more.

Ant assemblages tended to be distinct between the two aspects, and altitude and the first component of horizontal vegetation structure was also significant (global test statistic = 57.8, resid. df = 123; p < 0.001; Table 2, Fig. 2). Cluster analysis of β_sor_ (Electronic Supplementary Material Fig. 5) shows that communities stayed within their altitudinal ranges over time. At lower elevations (i.e., < 1530 m), ant assemblages were distinct between aspects, with assemblages found on the northern aspect quite different to those found on the south. Within those aspect groups, species composition also varied with elevation, but more markedly on the southern aspect, particularly for species above 1170 m (Fig. 2). At the highest elevations, however, aspect no longer delineated a difference between ant assemblages, with high altitude assemblages being significantly different from those at lower altitudes on either aspect (Fig. 2).

**Table 2:** Results of mvabund generalised linear model for pitfall traps grouped by altitude, aspect and sampling date.

	Residual degrees of freedom	Df.diff	Deviance	*p*
Intercept	107			
Altitude	106	1	1198.8	0.001
Aspect	105	1	606.8	0.002
Pch1	104	1	374.7	0.001

### Functional diversity (sesFD) across an altitudinal gradient

The best models explaining functional differentiation amongst ant species across the altitudinal gradient over the 6 years of our study did not include altitude, but included aspect and other factors associated with fine-scale habitat measures, such as horizontal and vertical vegetation structure, mean or minimum temperature. One model included season, and another included a measure of soil (Table 3). The best model obtained from model averaging found that sesFD was significantly lower on the southern aspect relative to the north, and was higher with increasing mean and minimum temperatures; however, the microclimate provided by vegetation in this setting also appears to be key: functional differentiation decreased with pch1 and pcv2, in other words, functional diversity increased with higher vegetation cover in the lower stratum and decreased when vegetation structure at higher strata became more complex. sesFD also increased with soil sandiness. The best-averaged model was:

$$sesFD \, = \, - 1.32 \, - 0.37 \, South \, + \, 0.07 \, Mean \, temp \, {-} \, 24.4 \, pch1 \, {-} \, 16.3 \, pcv2 \, + \, 0.18 \, summer \, + \, 0.06 \, MinT \, + \, 0.45 \, pcsoil2$$

**Table 3:** Best general linear mixed models used in model averaging, explaining variation in functional differentiation of ant species across an altitude gradient, over the 6 years of this study within 2 AICc of the best model.

	Intercept	Aspect	Mean T	Min T	pch1	pcv2	Season	pcsoil2	df	logLik	AICc	Δ AICc	weight
Model 1	− 1.90	+	0.08		− 22.8	− 20.4			7	− 94.0	201.5	0.00	0.29
Model 2	− 1.62	+	0.06		− 25.9	− 16.4	+		8	− 94.2	202.0	0.52	0.23
Model 3	− 0.88	+		0.06	− 24.7				6	− 98.6	202.1	0.59	0.22
Model 4	− 0.74	+		0.05	− 23.7	− 8.6			7	− 94.9	202.8	1.29	0.15
Model 5	− 0.87	+		0.06	− 25.4			0.45	7	− 97.9	203.5	1.98	0.11

Weight is a measure of the relative probability that that particular model is the best of the candidate models reported.

**Figure 3:**
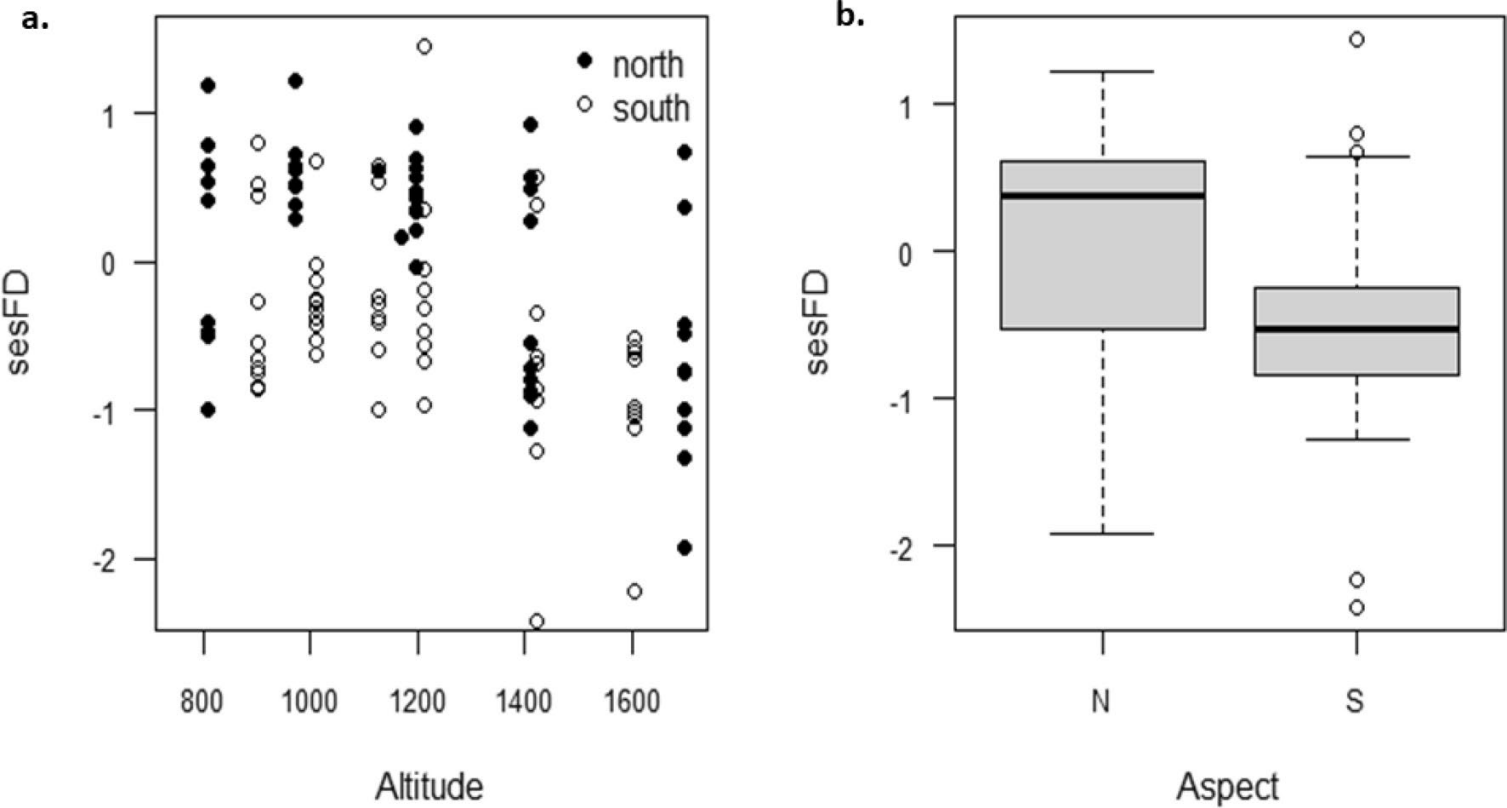
The relationship between sesFD and (**a**) altitude (not significant) and (**b**) aspect (significantly lower on the southern aspect, p < 0.05).

For the best model, fixed effects explained 39.8% of the variation, random effects (i.e., site) explained almost no extra variation. The worst model of those within 2 AICc of the best explained 38.6% of the variation, again, random effect explained almost no additional variation. A plot of aspect and altitude shows the variation in sesFD with altitude (not significant) and aspect (significantly lower on the southern aspect; Fig. 3).

## Discussion

Species composition change with altitude was driven primarily by species turnover, with a low contribution from nestedness. Thus each elevation has its own unique set of species, with the higher elevations, in particular, having unique sets of species. In addition to ant assemblages varying with both altitude and aspect, horizontal vegetation cover also influenced species composition (Table 2). We observed that the primary driver of species assemblages is altitude, which may be unsurprising, given that ectothermic species are more vulnerable to temperature fluctuations than endotherms, and species found at higher elevations occur in naturally isolated altitudinal niches^4,63^. Elsewhere, ant species occurring at higher altitudes have been found to occur at those elevations because they can tolerate cold temperatures better than more dominant species found at lower elevations either through physiology or behaviour^3,21^. Further assessment of the ability of ant species in this study to withstand temperature extremes would help to confirm or refute these findings, particularly if lower-altitude species are particularly cryophobic. Climate warming might pose a threat to these high elevation species, which could be outcompeted by species from lower elevations.

The highest sites on both sides of the mountain clustered together in the ordination, distinct from those at lower elevations on either side of the mountain. In the ordination, ant assemblages at middle and lower elevation sites also showed some separation according to altitude, but within their aspects (Fig. 2; Table 2). Habitat structure differed considerably between the two aspects at all altitudes except at the highest sites, where the vegetation structure was similar between north- and south-facing slopes. That the highest sites clustered together could also be partly explained by proximity: sites at the top of the mountain were closer to each other than those lower down, resulting in less dispersal limitation.

Partitioning of beta diversity suggests that the species found at these higher elevations are distinct from those lower down, and not a subset of lower elevation assemblages. This is of particular concern within the context of global climate change as this subset of species could go extinct. This is further emphasized by the decrease in nestedness with elevational distance, highlighting the uniqueness of these higher elevation sites. Over the 6 year period, assemblages do not seem to have changed, except perhaps those at higher elevations. Six years is a relatively short period over which to observe changes in species assemblages, and this explains the low explanatory power of time (r^2^ = 0.01). Nevertheless, this trend was significant and raises flags for the importance of monitoring high (and low) altitude assemblages over the long term.

### Patterns of functional diversity across an altitudinal gradient

Interestingly, although species composition was well explained by altitude, the functional diversity of those species was not. This suggests that as species composition changes with altitude, the functional traits represented amongst these species responds to other environmental factors, in this case, primarily habitat structure, along with temperature and soils.

Overall trends in FD across ecological gradients are beginning to emerge in the literature, although patterns vary^33,36,39,64^. For ant assemblages at altitude, our models found that FD (measured as sesFD; Fig. 3, Table 3) was lower on the southern aspect, but did not show a clear pattern with altitude. FD did vary with mean and minimum temperature, which vary with altitude, but also with habitat structure. Habitat structure, measured as horizontal and vertical structure emerged as significantly related to sesFD. As horizontal habitat structure linked to vegetation cover, increased, so too did sesFD (because pch1 was negatively correlated with horizontal habitat structure). Yet sesFD was negatively correlated with pcv1, which was a measure of vertical habitat structure, particularly that at > 50 cm height. Thus, it seems as if horizontal structure at low levels creates habitat complexity for ground-dwelling ants, so increasing functional differentiation (sesFD), but plant cover over 50 cm does not contribute to habitat structure, but may serve only to reduce ground temperature, which would be expected to be negative for ectothermic species. Plant cover above 50 cm in height is associated with increased shading and woody thickening, and is associated with relatively little ground cover. The implications of our findings are that studies focused on large scale measures like altitude may risk missing fine-scale changes associated with heterogeneous habitats. Habitat structure buffers changes in temperature and models based on temperature alone could overestimate the impact of climate change^65,66^. Fine-scale data collection is often laborious and costly, yet these data may make valuable contributions to large-scale models that would otherwise overlook microclimatic effects^23^. Adaptive Dynamic Global Vegetation Models can be used to simulate state variables causally linked to these fine scale variables^67^. These models also suggest that there could be a considerable lag in how vegetation responds to climate change, further confounding models based on temperature alone^68^. The importance of microclimate in dictating fine-scale biodiversity is increasingly recognised^9,69–72^. Here, we find that FD is well explained by finer-scale measures of habitat.

That FD, and to some extent species composition, are influenced by fine-scale factors implies that predicting species’ responses to climate change is complicated by habitat structure and may not be well predicted by only broad-scale predictors such as temperature and elevation^66^. This has implications for management: if vegetation structure can be maintained and managed, ectothermic species may be less impacted than expected, and it may be possible to ameliorate some of the inexorable effects of climate change, with careful conservation strategies. At the same time, drought intensity and frequency are predicted to increase^73,74^, with a risk that precipitation-dependent vegetation may die off.

At the regional scale, our findings shed light on the relative importance of high-altitude sites in Afromontane systems, which harbour distinct species assemblages. Vhembe Biosphere has recently received calls for all areas of high altitude to be declared core conservation zones, and our findings support this directive. Given that altitude is a major predictor of assemblage composition, as macro-climate warms, species should move upslope, as organisms attempt to remain within ambient temperatures to which they are adapted^6^. However, given that the climatically coolest areas are at the apex of mountains, such zones are by definition small in area. Therefore, with gradual movement up a gradient in response to warming, available habitat for a given species will likely shrink incrementally over time, ultimately jeopardising persistence^6^. Species at the lowest elevations may not be replaced, but lowest elevation communities may simply suffer species attrition^75^. Here, we found that ant species assemblages at each elevation are distinct, that nestedness makes very little contribution to beta diversity. In the context of global change, microclimates may be key to modulating the potentially deleterious impacts to high-altitude processes at a range of scales.

## Supplementary Information

Supplementary Information

## Data Availability

Intended archive: ResearchGate.
