# Supplementary material for: Repeated surveying over 6 years reveals that fine-scale habitat variables are key to tropical mountain ant assemblage composition and functional diversity
Source: Sci Rep. 2021 Jan 8;11:56. doi: 10.1038/s41598-020-80077-8 (PMC7794360; doi:10.1038/s41598-020-80077-8)
Supplement: Supplementary file 1 — Supplementary Information [file 41598_2020_80077_MOESM1_ESM.docx]

**Electronic supplementary material**

**Repeated surveying over six years reveals that fine-scale habitat variables are key to tropical mountain ant assemblage composition and functional diversity**

Mulalo M. Muluvhahothe ^a^ Grant S. Joseph ^a, b*^, Colleen L. Seymour^b, c^, Thinandavha C. Munyai^d^ and Stefan H. Foord ^a^

*^a^ SARChI-Chair on Biodiversity Value and Change, Department of Zoology and Centre for Invasion Biology , School of Mathematical and Natural Science, University of Venda, Private Bag X5050, Thohoyandou, 0950 South Africa*

*^b^Percy FitzPatrick Institute of African Ornithology, DST/NRF Centre of Excellence, Department of Biological Sciences, University of Cape Town, Rondebosch, 7701*

*^c^ South African National Biodiversity Institute, Kirstenbosch Research Centre, Private Bag X7, Claremont, 7735, South Africa*

*^d^School of Life Science, College of Agriculture, Engineering and Science, University of KwaZulu-Natal, Private Bag X01, Scottsville, 3209, South Africa*

*Corresponding author: Grant Joseph, email karoogrant@gmail.com

**
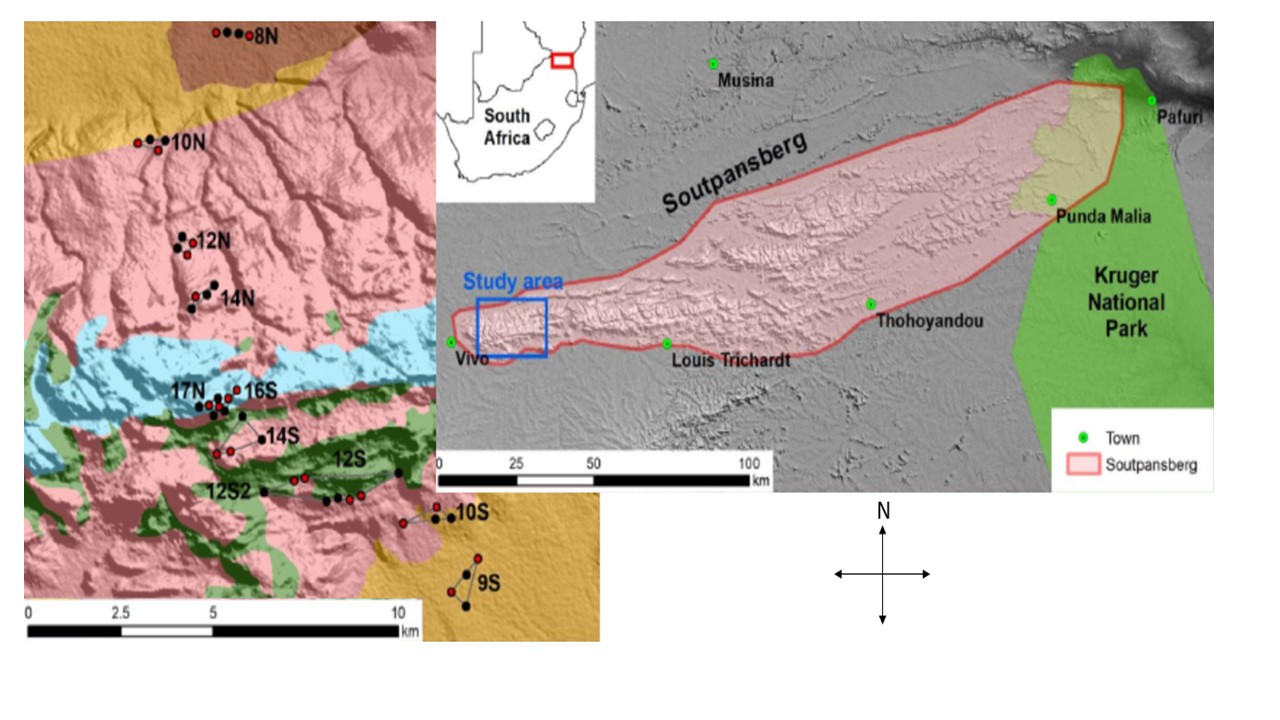
**

**Electronic Supplementary Figure 1.** Eleven sites spanned an elevational transect beginning at 23°02$'$16.91"S, 29°26$'$34.22"E, running north to south from 800 m above sea level (a.s.l.) on the southern aspect, ascending to 1700 m a.s.l., before descending to 800 m a.s.l. on the northern aspect. Map made by Norbert Hahn.


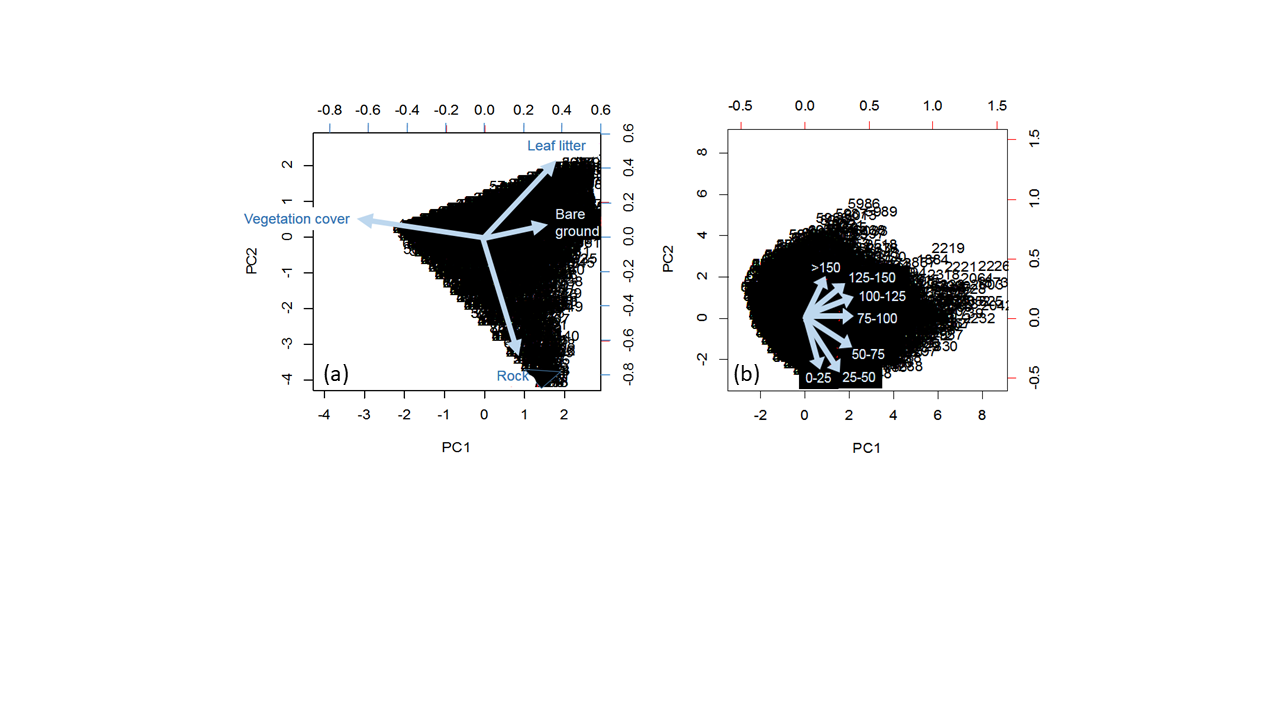


**Electronic Supplementary Information Fig 2**. PCA biplot for the horizontal habitat structure quantified for all surveys (a), and PCA biplot of the centroids for the 40 hits measured at each plot for vertical habitat structure for 15 surveys (b).


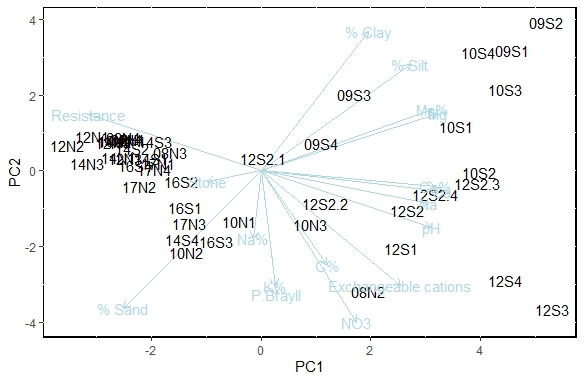


**Electronic Supplementary Figure 3.** PCA biplot for the soil components all survey sites.


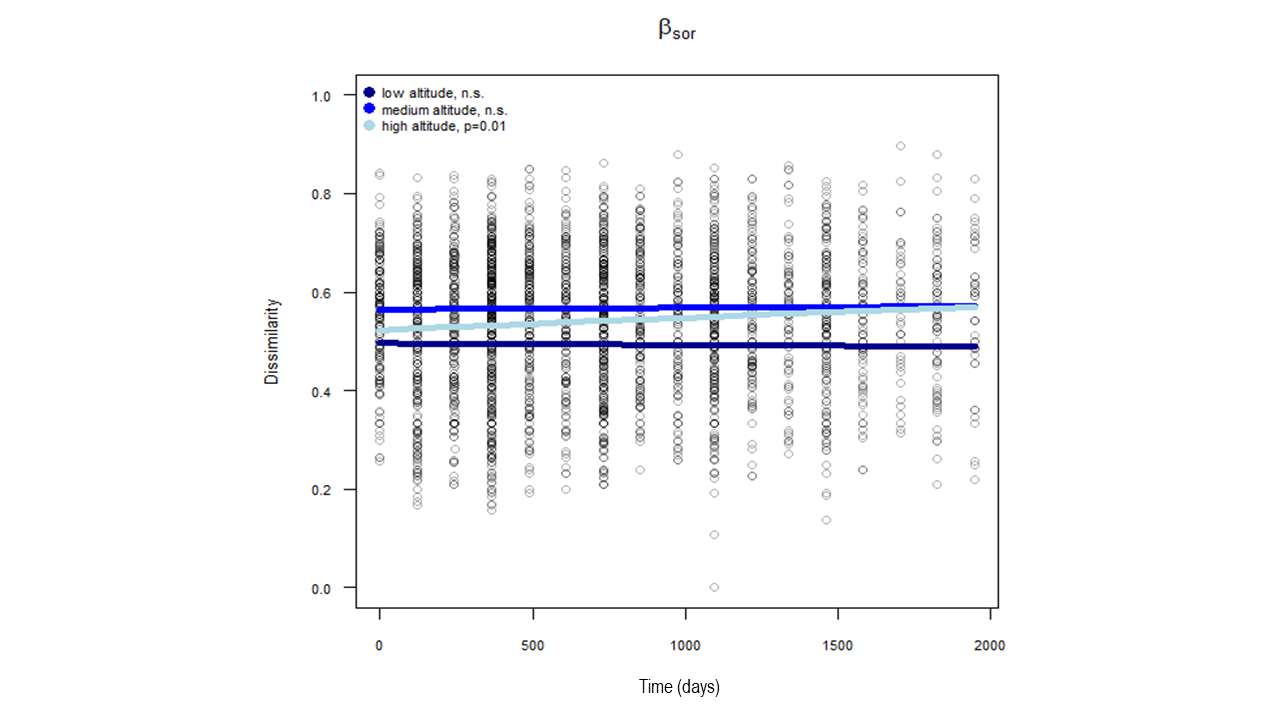


**Electronic supplementary figure 4.** Decay over time in total similarity (measured using Sorensen index, β_sor_) between ant assemblages at low, medium and high altitudes (both north and south aspects combined). Each data point represents a comparison between the first date of sampling and subsequent dates. There was no significant pattern of decay at low or medium altitudes, but there was a significant, although small, change over time for high altitude assemblages (p < 0.01).


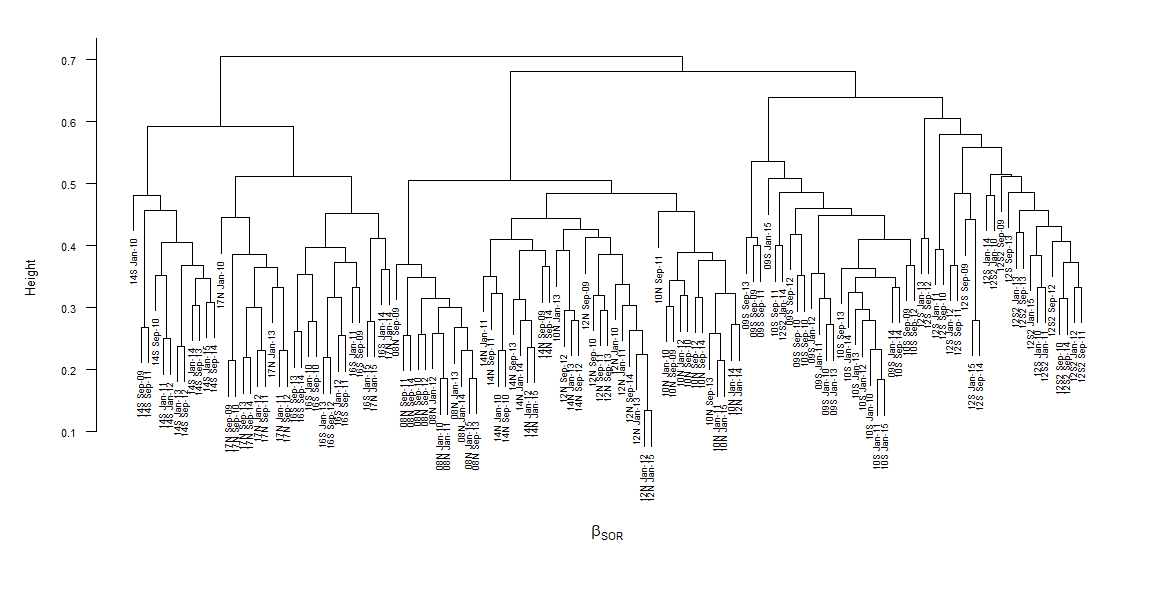


**Electronic Supplementary Material Figure 5.** Dendrogram showing relationships between sites at different dates of sampling, as measured by Beta Sorensen index.

**Supplementary Table 1.** Michaelis-Menton (MM) estimation of species richness (SR) for each set of sites at each altitude on each aspect based on data pooled for each six months’ sampling effort, for the penultimate and final six months of sampling.

| **Altitude & Aspect** | **SR MM: penultimate six months of sampling** | **SR MM: final six months of sampling** |
| --- | --- | --- |
| 8N | 42.2 | 42.5 |
| 9S | 54 | 54.3 |
| 10N | 48.4 | 48.8 |
| 10S | 47.4 | 47.9 |
| 12S | 48 | 48.2 |
| 14S | 54.1 | 54.8 |
| 14N | 54.6 | 55.1 |
| 16S | 48.2 | 48.8 |
| 17N | 40.5 | 41.1 |
